# Catechins and Their Effect on the Gut Microbiome in Health and Cancer

**DOI:** 10.1002/ptr.70435

**Published:** 2026-09-03

**Authors:** Prakash Monika, Bindu Sadanandan, Honnamachanahalli Ramachandraiah Tejashree, Maitreyi Darbha, Kirti Gupta, Srinivas Reneeka

**Affiliations:** ^1^ Department of Biotechnology M. S. Ramaiah Institute of Technology Bengaluru India

**Keywords:** cancer, catechins, EGCG, gut dysbiosis, gut microbiome, gut microbiota

## Abstract

Catechins are a group of flavonoids found primarily in green tea, fruits, and berries; they play a significant role in the modulation of the gut microbiota, thereby having an impact on gut health and cancer prevention. Owing to their low bioavailability, catechins are transformed by the gut microbiota into various bioactive metabolites such as valerolactones and glucuronides, which support the growth of several beneficial bacterial species including *Bifidobacterium* and *Lactobacillus* while inhibiting harmful strains like *Clostridium difficile* and *Escherichia coli* . These interactions enhance metabolic balance, reduce inflammation, and help maintain gut homeostasis, which is critical for preventing gut dysbiosis. Dysbiosis is characterized by a shift from symbiotic to pathogenic microbial populations and is closely associated with cancer development, especially in the gut. This review explores how catechins, particularly epigallocatechin gallate (EGCG), contribute to cancer prevention by promoting a symbiotic microbial environment, supporting immune modulation, and inhibiting inflammatory pathways. By examining the role of catechins in both health and cancer, this paper aims to emphasize their therapeutic potential as dietary supplements in maintaining gut health and reducing cancer risk.

## 1 Introduction

Polyphenols have garnered considerable attention in recent years for their wide‐ranging health benefits. They are abundant in a wide range of dietary sources, including fruits, vegetables, and grains. Initially, these compounds present challenges in terms of absorption; however, following metabolism by the gut microbiota, they are transformed into bio‐accessible metabolites that significantly enhance their pharmacological effects against various diseases (Gade and Kumar 2023). This metabolic transformation underscores the importance of dietary polyphenols and has led to new approaches in dietary supplementation, focusing on polyphenol‐rich foods like vegetables, fruits, especially berries, certain types of tea, and whole grains for promoting health and preventing disease.

Among the different types of polyphenols, flavonoids such as catechins are particularly noteworthy. These plant‐based secondary metabolites play a crucial role in managing both biotic and abiotic stress, while exhibiting potent free radical scavenging and antioxidant properties (Ullah et al. 2020). Catechins, characterized by their aromatic rings similar to resorcinol and catechol, possess five hydroxyl groups in their structure. This unique structure is responsible for their diverse physiological effects, including anti‐diabetic, anti‐bacterial, and most notably, anti‐cancer activity (Chen et al. 2019; Kim and Heo 2022). A balanced gut microbiome is essential in facilitating these benefits.

Catechins are known to promote the growth of probiotic microorganisms, including *Lactobacillus*, *Bifidobacterium*, and *Akkermansia*. These microbes play a key role in reducing inflammation, enhancing gut health, and modulating the microbiota (Hodges, Zhu, et al. 2020; Liu, Bruijn, et al. 2020; Pérez‐Burillo et al. 2021). The interrelationship of catechins and the gut microbiota is intricate and multifaceted. In healthy individuals, the consumption of catechins has been linked to an increased diversity of gut microbiota, which in turn has been associated with numerous health benefits (Filosa et al. 2018; Hervert‐Hernández and Goñi 2011). For instance, an enriched gut microbiota can enhance the bioavailability of catechins, leading to more effective prevention of various diseases (Bié et al. 2023). Many studies have focused on understanding how catechins are metabolized by the gut microbiota and how these metabolites affect the microbiome. However, despite these promising findings that catechins have a positive impact on gut health, the exact pathways and mechanisms involved are not fully understood and warrant further investigation (Cueva et al. 2020; Huang et al. 2013).

Alongside their impact on the healthy gut microbiome, catechins have also been extensively researched for their potential in the prevention and treatment of cancer. Colorectal cancer, in particular, has been of major interest due to its high prevalence and mortality rate (Oh et al. 2023). Studies have shown that catechins possess the capability to prevent the invasion and metastasis of colorectal cancer which could serve as a valuable adjunct in colorectal cancer treatments (Chopra et al. 2022). However, the association between gut microbiota, catechins and cancer is multifaceted, with many confounding factors that make it difficult to draw definitive conclusions. The gut microbiota itself is influenced by numerous factors, including diet, lifestyle, and genetics, all of which can affect the metabolism and anti‐cancer effects of catechins (Piao et al. 2017).

Studies have also highlighted the potential of catechins in modulating immune responses, which could have a substantial impact on cancer prevention. The anti‐inflammatory properties of catechins are particularly relevant in this context. By reducing chronic inflammation, which is an established risk factor for cancer, catechins may help lower the incidence of cancer. Additionally, catechins have been shown to modulate various signaling pathways involved in cancer development and progression (Cadoná et al. 2022).

Despite these encouraging findings, a considerable gap persists in the literature concerning the precise mechanisms through which catechins exert their effects on both the healthy gut microbiome and cancer (Ding et al. 2020). Moreover, while in vitro and animal studies have provided valuable insights, the translation of these findings to humans remains challenging. As a result, additional research is necessary to elucidate the role of catechins in health and disease, especially concerning the gut microbiome and cancer.

In this review, we have comprehensively examined the epimers of catechins such as (−)‐Epicatechin (EC), (+)‐Catechin, (−)‐Epigallocatechin (EGC), (−)‐Epicatechin gallate (ECG), and (−)‐Epigallocatechin gallate (EGCG), elucidating their biological effects and metabolism in the gut microbiota. We have specifically focused on their role and mechanisms of action during health and cancer, highlighting the differential impact and potential therapeutic implications of catechins in these distinct physiological contexts.

## 2 Catechins and Their Source

Catechins, primarily polyphenolic compounds, are a subgroup of flavonoids found in excess concentrations in plant‐derived fruits, beverages, and vegetables (Gadkari and Balaraman 2015). With a ring and the elementary structure of flavan‐3‐ol, catechins are condensed tannins, containing hydroxyl groups (—OH) in their chemical structure that bind with other materials (Bae et al. 2020). Two aromatic rings and a heterocycle ring with a gallate moiety (—OH) at the C‐3 position constitute the basic structure of catechins (Pedro et al. 2020). Based on their chemical structure and depending on the substitution of the gallate moiety at the C‐3 position, they are classified into gallated and non‐gallated catechins (Fan et al. 2016). The four most important classes are EC, ECG, EGC, and EGCG (Bae et al. 2020). The chemical structure of EC consists of three hydrocarbon rings having six hydroxyl groups at different sites, influencing its hydrophilic nature (Liu et al. 2023). Figure 1 illustrates the chemical structure of catechin and its types. EGC is similar to EC in chemical structure, except for the presence of the trihydroxyl group (Han et al. 2023). EGCG and EGC are akin to each other in chemical structure, with the key difference being the absence of gallic acid ester in EGC. Both compounds retain the trihydroxyl group, with EGCG constituting a benzenediol ring connected to a tetrahydrophan moiety, a pyrogallol ring, and a galloyl group. It forms a four‐ringed structure with eight hydroxyl groups contributing to its high hydrophilicity (Furniturewalla and Barve 2022).

**FIGURE 1:**
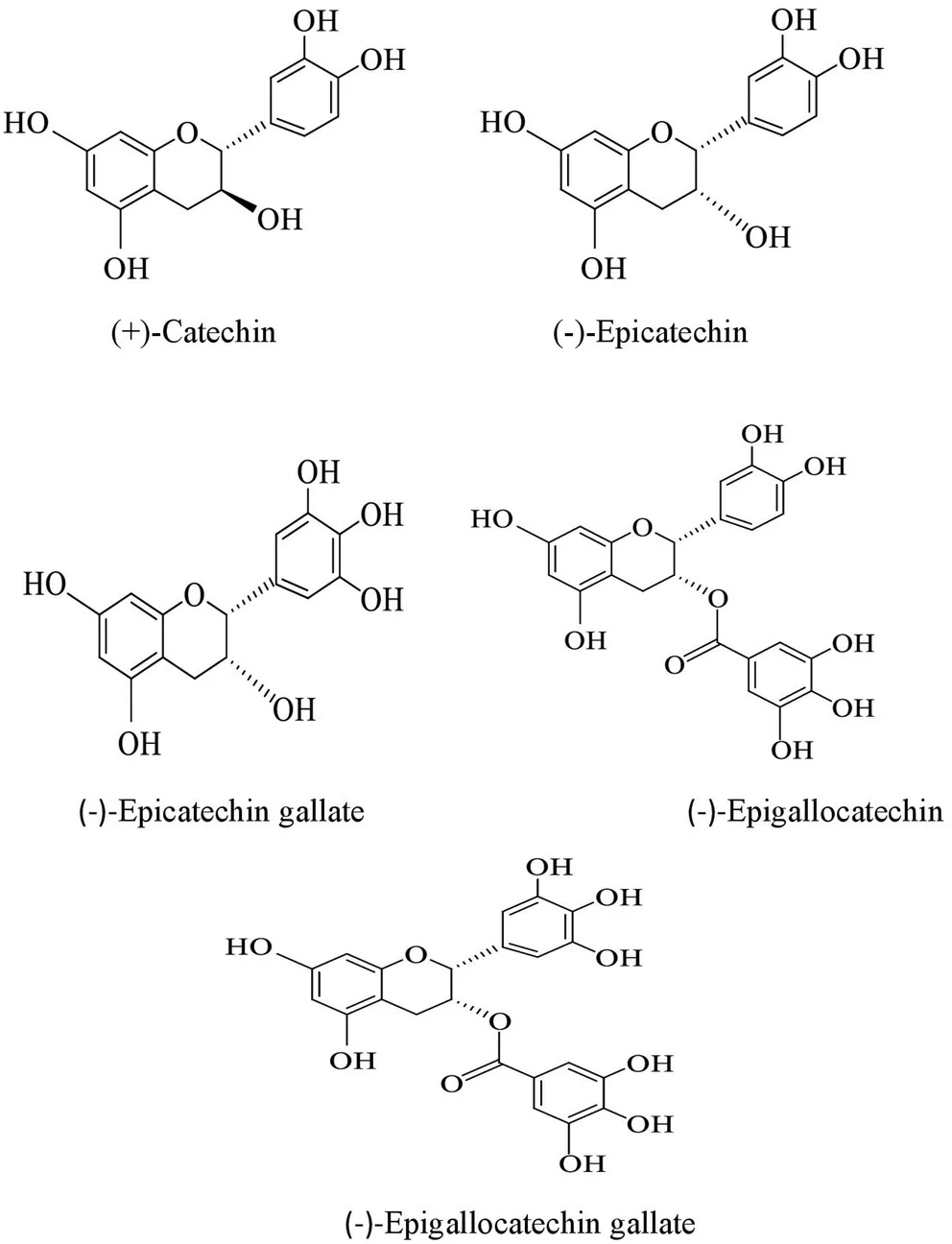
Catechins and their chemical structure.

Camellia species, especially, *Camellia sinensis* and *Camellia assumica* are reported to be one of the major sources of catechins (Ambigaipalan et al. 2020; Bae et al. 2020). Catechins are abundantly present in apricots, strawberries, red wine, cocoa, pomegranate juice, fresh tea leaves broad beans, and black grapes. ECs are present in high amounts in apples, blackberries, raspberries, dark chocolate and argan oil (Cione et al. 2019; Frasheri et al. 2020). EGC, ECG, and ECGC are present in comparatively small quantities in fruits such as berries, black currants, and grapes. Green tea is considered as the most important source of catechin comprising 40% of the overall phenolic content, followed by black tea and oolong tea (Fan et al. 2016; Trisha et al. 2022). Also, species of *Allium, Brassica*, and *Lactuca*, primarily onions, broccoli, and lettuce are good sources of catechins. (Gadkari and Balaraman 2015). Table 1 provides a comprehensive overview of different sources of catechins, highlighting their specific catechin compositions.

**TABLE 1:** Sources of catechin and their composition.

Sl. no.	Sources	Other catechins	EC	ECG	EGC	EGCG	References
1	Purple tea leaves	1.02%	0.5%	2.80%	1.6%	11.9%	(Abdel‐Aal et al. 2022)
2	Tomato	0.02%	0.002%	—	0.003%	—	(Gürbüz Çolak et al. 2020)
3	Cashew nut testa	4.72%	2.82%	—	—	0.2%	(Sruthi and Naidu 2023)
4	Greek Oregano	0.014%	1.967%	—	—	—	(Kosakowska et al. 2021)
5	Cyprus Carob pulp	0.047%	0.011%	—	—	—	(Ioannou et al. 2023)
6	Matcha Organic	0.19%	0.5%	—	2.4%	6.9%	(Meyer et al. 2023)
7	Airen Grapes	0.006%	0.003%	—	—	—	(García‐Martínez et al. 2021)
8	Lintongsuan Pomegranate	0.041%	0.035%	—	—	—	(Pirzadeh et al. 2021)
9	Royal Gala Apple	0.0089%	0.0095%	—	—	—	(Preti and Tarola 2021)
10	Avocado peel	0.061%	0.152%	—	—	—	(Restrepo‐Serna and Cardona‐Alzate 2024)
11	Guara Spanish almonds	0.166%	0.133%	—	—	—	(Moreno Gracia et al. 2021)
12	Black tea	0.3%	0.31%	—	0.83%	0.51%	(Deo et al. 2024)
13	Searless Cranberry	0.428%	1.207%	—	—	—	(Gudžinskaitė et al. 2020)
14	Coffee	0.479%	—	—	—	—	(Steger et al. 2022)

## 3 Properties of Catechins

### 3.1 Physical Properties

The physiochemical properties of catechins directly influence their stability, solubility, and bioavailability, aiding their interactions with the gut microbiota. As polyphenolic compounds, they have an unpleasant taste and appear as pale yellow to colorless crystalline substances in their pure form (Gadkari and Balaraman 2015). The molecular structure and molecular weight (290 g/mol) significantly influence catechin's integrity to assist in biological efficacy (Ahmadi et al. 2020). They exhibit poor water solubility but are highly soluble in organic solvents like methanol and ethyl acetate, the latter being used to extract catechins from plant sources. Their stability is highly sensitive to light, oxygen, humidity, temperature, and pH, making it necessary to stabilize them in the final formulation (Malrianti et al. 2021; Ye and Augustin 2019). EGCG is found to be unstable in pure form and retains much of its bioactivity at neutral pH of around 7.2 and a melting point of roughly 218°C. Studies reveal a direct correlation between solubility and temperature in EC, as indicated by an increased dissolution across pure solvents under an elevated temperature range of 148°C–170°C (Peter et al. 2017). Whereas ECG is relatively stable in acidic pH, in the range 2.0–4.0 and temperatures above 130°C (Zhu et al. 2023). Additionally, catechins are characterized, isolated, and quantified by High Pressure Liquid Chromatography, various spectrophotometric methods namely UV‐Spectrophotometry, Hydrogen and Carbon Nuclear Magnetic Resonance Spectrophotometry, and High‐Resolution Magnetic Spectroscopy (Ahmad et al. 2014; Gadkari and Balaraman 2015; Kalai Selvi and Nagarajan 2018). The absorption maxima for EC, ECG, EGC, and EGCG were observed within the wavelength range of 210–280 nm (Musial et al. 2020).

### 3.2 Chemical Properties

The molecular structure of catechin contributes to various parameters such as degradation, oxidation, and epimerization. These influence the release kinetics profile and subsequent engagement with biomolecules, enhancing the bioactivity. Catechin's ability to interrelate with other compounds facilitates hydrogen bonding and protein interactions.

Epimerization is the process by which the configuration of catechins changes. The conversion of EGCG to gallocatechin gallate occurs within a temperature range of 25°C–100°C (Chen et al. 2021). At temperatures between 60°C and 90°C, up to 70% of EC is converted to catechin, with more hydroxyl substitutions on ortho and para positions. At temperatures around 200°C, catechins begin to degrade rather than undergoing simple structural arrangement, thereby impacting their bioactive properties at elevated temperatures (Fernández‐Romero et al. 2020; Liu, Le Bourvellec, et al. 2021). The presence of ortho hydroxyl groups in catechol ring contributes to its strong antioxidant and electron‐donating properties (Anitha et al. 2022). Oxidation of catechins follows a similar trajectory, but is more dependent on pH and on environmental factors like oxygen availability. Studies report that in EGC a gradual change in the color from colorless to brown was observed when heating temperature was increased to 60°C for a duration of 40 min; however, acidic conditions slow down the oxidation and degradation process (Zhu et al. 2023). EC is observed to be unstable under blue light illumination, undergoing a photosensitive redox reaction to generate superoxide radicals (O_2_˙^−^). Conversely, on addition of EGCG to the compound containing EC, the former suppresses the photosensitive oxidation at pH below 4.0 (Huang et al. 2019). Therefore, these processes are codependent, and the reactions can alter the stability and formulation; they also provide insights on its application in food and biomedical sciences.

### 3.3 Biological Properties

Catechins provide a varied range of biological properties, primarily anticancer, anti‐inflammatory, and antioxidant. These properties aid in modulating cellular pathways and neutralizing toxins in the body. In this section, the biological properties are briefly outlined, emphasizing their potential in antioxidant, anti‐inflammatory, and anti‐tumor activities (Luo et al. 2020; Pedro et al. 2020; Anitha et al. 2022).

#### 3.3.1 Antioxidant Activity

Catechins demonstrate strong anti‐oxidant properties due to their one‐electron reduction potential (Kim et al. 2022). Research reveals that the ortho‐dihydroxy configuration of the catechol B‐ring and the galloyl group at C‐3 position undermines the radical scavenging activity of catechins. They neutralize reactive oxygen species, chelate pro‐oxidant transition metals and upregulate endogenous antioxidant enzymes such as catalase and glutathione peroxidase (Pedro et al. 2020; Prakash et al. 2019). Additionally, inhibition of redox active transcription factors, reduction in nonheme iron, plasma lipid peroxide and malondialdehyde leads to simultaneous increase in plasma antioxidant activity (Bernatoniene and Kopustinskiene 2018). In comparison to glutathione, vitamin C, and other flavonoids, catechins show better antioxidant activity, making them a functional material in amending human health and cellular redox homeostasis (Kim and Heo 2022).

#### 3.3.2 Anti‐Inflammatory Activity

Inflammation serves as a critical defense mechanism within the body, essential for combating pathogens and initiating the healing process. Catechins possess anti‐inflammatory properties, easing liver steatosis, injury, and activating NF‐κB pathways in both genetic and diet‐induced models of nonalcoholic steatohepatitis (Hodges, Sasaki, and Bruno 2020). ECG mitigates the inflammatory response by preventing the excessive synthesis of inflammatory mediators in vascular smooth cells (Li, Feng, et al. 2022). Interestingly, EGCG reduces gut mucosal inflammation by suppressing the production of NF‐κB, TLR4, and My88 signaling pathways, alleviating inflammatory diseases (Sharifi‐Rad et al. 2020). Similar findings were concluded by Kim et al., where in vivo studies conducted confirmed that when EGCG is administered, it effectively blocks the RANKL/RANK signaling pathway (Kim et al. 2020). This pathway has been shown to have a crucial role in the activity and survival of mast cells involved in inflammation. Studies reveal that nitric oxide production in mouse Kuppfer cells was inhibited by catechins, simultaneously decreasing the expression of nitric oxide synthase in lipopolysaccharide cells (Kim et al. 2021).

#### 3.3.3 Anti‐Tumor Activity

Catechins are proven to induce cell death and anticancer activity in prostate cancer, breast cancer, and lung cancer. ECG is reported to have anti‐cancer activity in the cancer initiation and promotion phases. It inhibits the arachidonic acid metabolism, thereby preventing inflammation‐induced carcinogenesis in colon mucosa as well as in colon tumors (Cheng et al. 2020). ECG modulates the molecular expression during carcinogenesis through its interaction with various molecules, including enzymes, transcription factors, and signaling molecules, making it an ideal chemo preventive agent. In initiation, it inhibits Phase I enzyme CYP450, suppressing abnormal cell growth. Whereas in the progression stage, it induces apoptosis by increasing the expression of p53, a critical cell‐cycle regulator (Li, Feng, et al. 2022). Additionally, ECG and EGC, in significantly less quantities, have been associated with anti‐proliferative and antiangiogenic activity (Oh et al. 2023). EGCG inhibits the metalloproteinase activity in the extracellular matrix of the tissues, inducing apoptosis and arresting cell cycle in colon cancer HCT‐116 cells. It is able to trigger both intrinsic and extrinsic apoptotic pathways, including caspase‐3 activation, nuclear condensation, cleavage of poly (ADP) polymerase, and inhibition of insulin‐like growth factor‐I (IGF‐I) (Musial et al. 2020). The ability of catechins to modulate cellular mechanisms and protection against oxidative stress gives them an edge over other phytocompounds in managing various health conditions.

## 4 Gut Microbiome in Healthy Individuals

A microbiota is a distinct community of microorganisms comprising of bacteria, some single‐celled eukaryotes, and archaea. Conversely the microbiome is made up of all the genetic components of a microbiota (D'Argenio and Salvatore 2015). To define the state of gut microbiome in a healthy individual, it is necessary to understand the composition of gut microbiota. The gut microbiota cataloged as a “vital organ” is estimated to account for more than five million different genes till date, with a thousand different species colonizing the human gut (Afzaal et al. 2022; Ruan et al. 2020). Approximately five million distinct genes are estimated in the adult gut microbiome with the major components being a thousand known species belonging to smaller phyla, *Firmicutes*, and *Bacteroidetes*, and some species of *Actinobacteria*, *Proteobacteria*, *Fusaobacteria*, and *Verrumicrobia* (D'Argenio and Salvatore 2015; Trakman et al. 2022).

The high microbial diversity is essential in maintaining the core microbiome, digestion of polysaccharides, protection against infections, development of immune system, behavior, and controls angiogenesis (D'Argenio and Salvatore 2015). Apart from this, the microbiome plays a vital part in the initiation of microbial metabolites that benefit microbes as well as the host, modulating both intra (microbe–microbe) and inter‐kingdom (microbe–human) interface (Ruan et al. 2020). Vitamins, essential organic molecules that perform metabolic functions, are another key product of gut microbiota (Trakman et al. 2022). Through microbial fermentation, specific species contribute to the production of metabolites such as Vitamin K2 (Menaquinones) and Vitamin B complex (folate, biotin, and riboflavin). These act as a source of energy, promoting fatty acid synthesis and gluconeogenesis (McBurney et al. 2019). This symbiotic relationship benefits the host's metabolic needs, disruption of which can lead to alteration in the metabolic pathway, queuing the onset of diseases like cardiovascular disease and non‐alcoholic fatty liver. Notably, antibiotics supplementation often worsens gut health in such cases, while the introduction of polyphenols and natural substances in the diet has proven to be beneficial in restoring balance.

## 5 Effect of Catechins on Healthy Human Gut Microbiome

Diet plays a pivotal role in initiating, modulating, and shaping the gut microbiota composition and diversity which greatly influences overall health. Gut bacteria can metabolize dietary fibers and polyphenols found in various foods to produce SCFA and other metabolites which are known for their health‐promoting properties, particularly in maintaining intestinal barrier integrity and reducing inflammation which are crucial for the prevention of gut microbiota‐mediated chronic diseases. However, the ability of the gut microbiota to produce these metabolites varies between individuals due to various factors including age, diet, environment, and genetics. This variation necessitates understanding of gut microbial activity which requires assessing the activity, composition, and total microbial number at different intestinal locations (Loo et al. 2020).

Catechins found abundantly in black tea, green tea, and oolong tea have gained attention for their ability to modulate gut microbiota. Studies show that tea catechins can increase the growth of beneficial bacteria such as *Lactobascillus‐Enterococcus* and *Bifidobacterium* both of which are involved in SCFA production, while limiting the growth of pathogenic bacteria like *Bacteroides‐Prevotella* and *Clostridium histolyticum* (Sun et al. 2018). In addition, black and green tea catechins have been observed to restrict the proliferation of harmful bacteria namely *Enterococcus faecalis* , *Escherichia coli* , *Pseudomonas aeruginosa* , and *Staphylococcus aureus* (Bancirova 2010). Catechins significantly promoted the growth of *Actinobacteria* and *Proteobacteria*, while inhibiting the growth of *Bacteroidetes, Firmicutes*, and *Fusobacteria*. This altered the microbiota composition, resulting in distinct microbial patterns (Huang et al. 2016). In an in vivo study conducted by Guo et al., green tea catechins significantly increased *Bacteroidetes* and *Proteobacteria*, while significantly decreasing the levels of *Firmicutes* (Guo et al. 2017). In addition, a human trial involving 12 healthy individuals (aged 24–36 years), the consumption of green tea enriched with catechins for 2 weeks resulted in increase in the populations namely *Blautia*, *Coprococcus*, *Dorea*, *Eubacterium*, *Feacalibacterium*, and *Roseburia* all of which are specialized in producing SCFA (Yuan et al. 2018). However, the number of studies investigating the influence of pure EC on gut microbiome composition remains limited. Initial research using human fecal samples has shown that EC can increase the growth of bacterial groups such as *Eubacterium rectale* , *C. coccoides*, known for producing beneficial SCFA (Tzounis et al. 2008). Further, in vitro treatment with EGCG has shown significant increase in the growth of beneficial bacteria such as *Bifidobacterium* and *Bacteroides*, while simultaneously inhibiting the proliferation of pathogenic bacteria such as *Enterobacteriaceae* and *Fusobacterium* (Liu, Bruijn, et al. 2020). Another in vitro study conducted by Zhang et al., on human intestinal microbiota reported that EGCG, epigallocatechin 3‐O‐(3‐O‐methyl), and gallocatechin gallate extracted from Chinese oolong tea promoted the growth of *Bifidobacterium* and *Lactobacillus–Enterococcus* species and suppressed the growth of *Bacteroides–Prevotella, C. histolyticum*, and *Eubacterium–Clostridium* groups. The total SCFA concentrations in cultures were also found to be higher (Zhang et al. 2013). Additionally, EGCG has been found to increase the growth of *Akkermansia*, an anti‐obesogenic bacterium (Liu, Zhao, et al. 2020; Pérez‐Burillo et al. 2021). Further in vivo studies examining the effects of catechins on the gut microbiome composition, as summarized in Table 2, highlight their potential to modulate microbial populations. In conclusion, many in vitro, in vivo, and few clinical studies demonstrate that catechins and their metabolites affect the gut microbiota composition by increasing beneficial micro flora and restricting the growth of pathogenic microbes, thereby positively influencing human health.

**TABLE 2:** In vivo studies demonstrating the effect of catechins on gut microbiome composition.

Sl. no.	Type of model	Effect on the gut microbiota composition	Key findings	References
1	Female C57BL/6J mice	Increased *Akkermansia, Bacteroides, Lactobacillus*	Green tea catechins enhanced intestinal epithelial homeostasis and also alleviated symptoms of experimental colitis	(Wu, Huang, et al. 2021)
2	Female db/db and C57BLKS/J mice	Increased *lactobacillus, Bifidobacterium* Decreased *Clostridium, Roseburia*	Green tea catechins altered the gut microbiota composition	(Wu, Liu, et al. 2021)
3	Male C57BL/6J mice	Increased *Biffidobacterium, Akkermansia, Ruminococcus* Decreased *Firmicutes*	Green tea extracts improved gut microbiota composition, reduced dysbiosis, improved intestinal barrier function which decreased adipose inflammation	(Dey et al. 2019)
4	Male Sprague Dawley rats	Increased *Parabacteroides, Phascolarctobacterium, Prevotella, Robinsoniella* Decreased *Ruminococcus Lachnospira, Oscillospira*	Catechin when supplemented with a fructo‐oligosaccharide diet, alters the cecal microbiota and affects the gene expression in colonic epithelial cells, leading to weight loss	(Luo et al. 2018)
5	Male C57BL/6N mice	Increased *Akkermansia, Allobaculum* Decreased *Desulfovibrionaceae*	EGCG improved gut microbiota composition, reduced dysbiosis and corrected dysregulation of serum bile acids	(Ushiroda et al. 2019)
6	Male C57BL/6 mice	Increased *Alloprevotella, Muribaculaceae, Bacteroides* Decreased *Blautia, Colidextribacter*	EGCG influenced the gut microbiota composition while modulating the gut‐brain axis to reduce obesity	(Zhou et al. 2023)
7	Mouse model with diabetes	Increased *Lactobacillaceae* Decreased *Lachnospiraceae, Ruminococcaceae*	EGCG and tea polyphenols significantly improved the gut microbiota composition in diabetic mice	(Liu et al. 2022)
8	ICR mice	Increased *Akkermansia, Helicobacter, Bacteroides*	EGCG stabilized gut microbiota and serum metabolites during acute stress induced by lipopolysaccharide	(Ma et al. 2021)
9	Male C57BL/6J mice	Increased *Lactobacillus gasseri* , *Lactobacillus murinus* , *Lactobacillus reuteri* , *Bacteroidales*, *Blautia, Turicibacter*	EGCG supplementation was found to modulate gut microbiota composition, offering protection against intestinal injury caused by radiation in mice	(Cai et al. 2022)
10	Male C57BL/6J mice	Increased *Bacteroidetes, Lachnospiraceae*, *Ruminococcaceae*	EGCG reduces DNA strand breaks and modulates DNA methylation of key genes like Dnmt1 and MLH1, preserving gut microbiota balance	(Remely et al. 2017)
11	Male C57BL/6J mice	Increased *Verrucomicrobia*, *Bacteroides*, *Parasutterella*, *Akkermansia* Decreased *Firmicutes*, *Proteobacteria*, *Tenericutes*, *Roseburia*	EGCG enhances ileal immune function by modulating the gut microbiome and increasing immunoglobulin production in mice on a high‐fat diet	(Liu, Zhao, et al. 2021)
12	Male Sprague Dawley rats	Increased *Bifidobacterium*, *Turicibacter*, *Faecalibaculum*, *Alloprevotella*, *Allobaculum* Decreased *Firmicutes*, *Desulfovibrio*, *Blautia*	The combined use of low doses of EGCG and caffeine exhibits a synergistic anti‐obesity effect by regulating gut microbiota composition and bile acid metabolism	(Zhu et al. 2021)

## 6 Mechanism of Action of Catechins on Gut Microbiota in Healthy Humans

Catechins exhibit broad physiological activity, contributing to gut and metabolic health in non‐diseased states. Their capacity to cross the blood–brain barrier and affect neuronal activity has been associated with mitigating neurodegenerative conditions, including Alzheimer's and Parkinson's diseases (Kim and Heo 2022; Unno et al. 2017). However, the bioavailability of catechins remains limited, as they undergo extensive metabolism in the gastrointestinal tract and liver, resulting in only a small fraction entering systemic circulation (Cai et al. 2018). Understanding the pharmacokinetics of catechins, particularly their absorption, distribution, metabolism, and excretion (ADME), is crucial to optimizing their therapeutic potential.

The ensuing section provides a comprehensive analysis of the pharmacokinetic profile of catechins, with an emphasis on ADME. Additionally, it addresses the pivotal function of gut microbiota in biotransforming catechins into secondary metabolites, which contribute to their immunomodulatory and anti‐inflammatory effects. By exploring recent findings in this domain, this section further elucidates the potential of catechins to exert profound physiological impacts despite their inherently low bioavailability.

### 6.1 ADME of Catechins

The ADME properties of catechins determines their physiological efficacy. After ingestion, catechins are absorbed primarily in the small intestine, with only a small fraction reaching systemic circulation. The majority pass into the colon, where gut microbiota metabolizes them into bioactive metabolites, such as glucuronides, sulfates, methylated derivatives, and phenolic acids. Phase I and II biotransformation, including oxidation, reduction, hydrolysis, and conjugation, plays a crucial role in this process. Upon absorption, catechins undergo rapid glucuronidation and sulfation, distributing metabolites into the bloodstream, where they exert antioxidant, anti‐inflammatory, and vasoprotective effects. The overall pharmacokinetic profile, including the absorption, distribution, metabolism, and excretion (ADME) of catechins in the body, is illustrated in Figure 2. Among these, EC demonstrates particularly efficient absorption, with up to 95% of the ingested dose excreted in urine and feces after Phase II metabolism in the gastrointestinal tract and liver (Ottaviani et al. 2016; Qu et al. 2021). However, Cai et al., reported notable discrepancies in the relative contribution of microbial versus hepatic metabolism. These variations are likely attributable to inter‐individual differences in gut microbiota composition, host genetics, and analytical methodologies used to quantify catechin metabolites (Cai et al. 2018).

**FIGURE 2:**
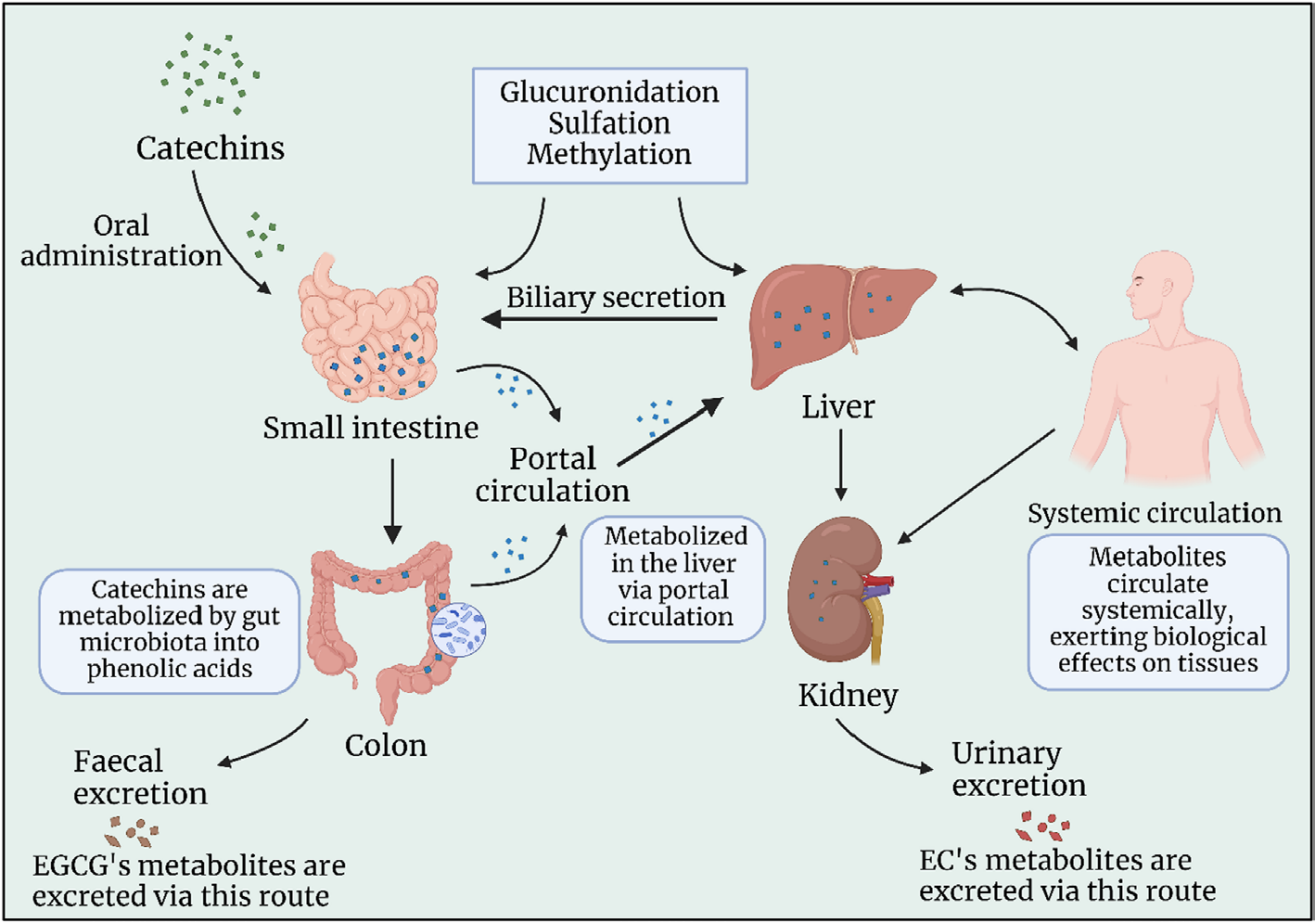
ADME of catechins in human body. This schematic outlines the journey of catechins from ingestion to excretion. After oral intake, catechins are absorbed in the small intestine and undergo initial metabolic transformations in the liver. In the colon, gut microbiota further convert these compounds into various metabolites. These metabolites then enter systemic circulation, exerting biological functions across different tissues and are eventually eliminated through the kidneys and colon. Key processes such as glucuronidation, sulfation, and methylation are essential for their biotransformation and effective transport.

Catechins undergo metabolic transformations through both host and microbial processes. Gut bacteria, such as *Clostridium coccoides* and *Eubacterium* sp. metabolize into smaller phenolic acids with distinct physiological roles. Non‐galloylated catechins like EC and catechin are metabolized through C‐ring opening and hydroxylation, resulting in metabolites like 3‐Hydroxyphenylacetic acid, which has anti‐inflammatory properties (Chen et al. 2020; Liu et al. 2018). Catechins modulate host immune balance by influencing microbial metabolites and regulatory T cell activity, helping maintain immune homeostasis and preventing excessive inflammation as depicted in Figure 3 (Guo et al. 2019; Vernocchi et al. 2020).

**FIGURE 3:**
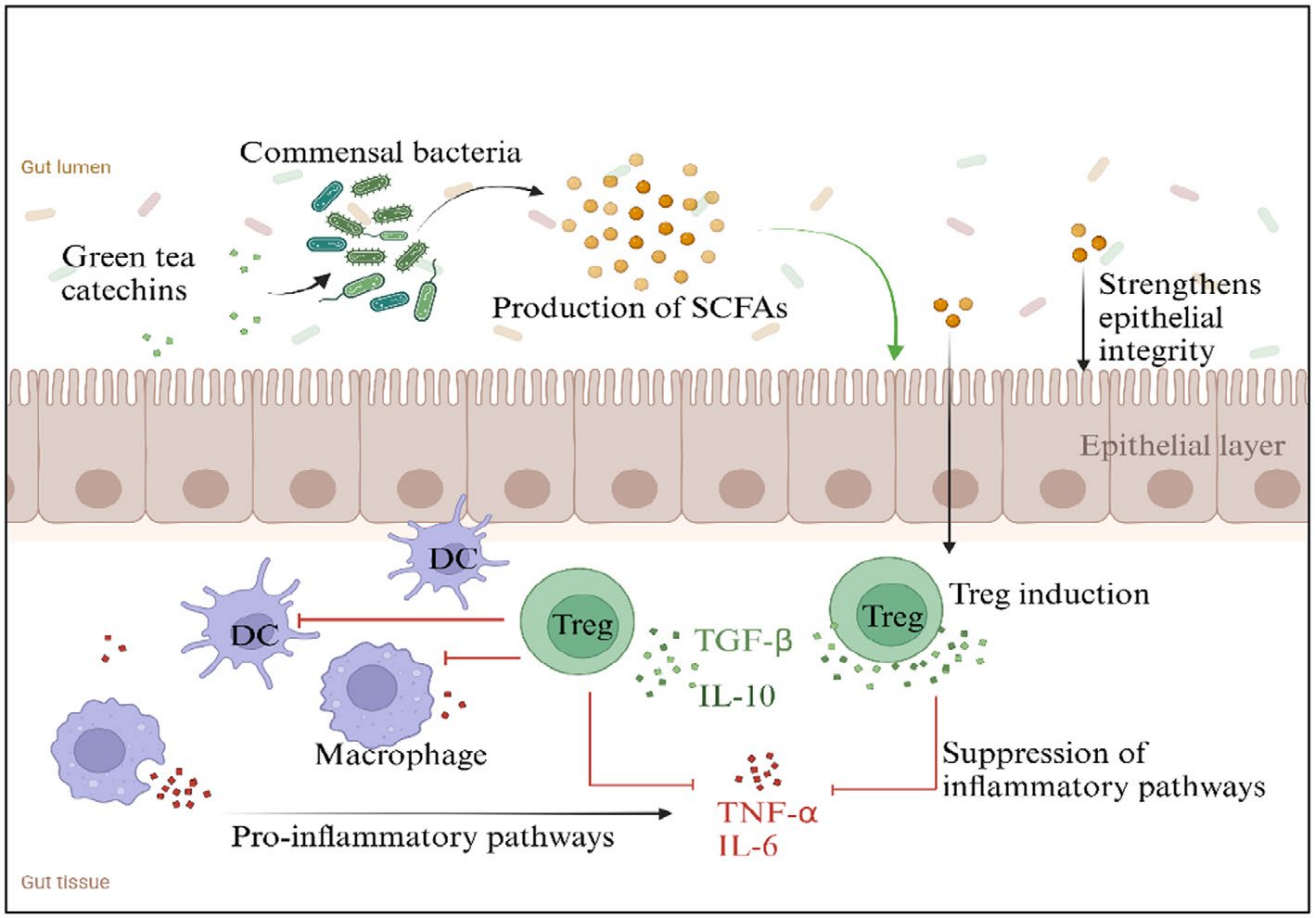
Mechanism of action of catechins on the gut microbiota under healthy conditions. This figure demonstrates the role of green tea catechins in modulating the gut microbiome and their subsequent effects on intestinal health. Upon ingestion, green tea catechins interact with commensal bacteria, stimulating the production of SCFAs, which are beneficial metabolites that help maintain epithelial barrier integrity. The enhanced epithelial barrier supports immune homeostasis, while SCFAs are pivotal in the induction of regulatory T cells (Tregs). Tregs, in turn, secrete anti‐inflammatory cytokines, including IL‐10 and TGF‐β, which suppress pro‐inflammatory pathways by inhibiting cytokines such as IL‐6 and TNF‐α. Additionally, dendritic cells (DCs) and macrophages, which are typically involved in pro‐inflammatory responses, are downregulated by the anti‐inflammatory actions of Tregs, promoting an overall immunoregulatory environment in the gut.

Galloylated catechins, such as ECG and EGCG, are hydrolyzed by microbial enzymes to release aglycone forms. EGCG, a potent catechin, exhibits antioxidant, anti‐inflammatory, and chemoprotective properties but has limited bioavailability due to first‐pass metabolism. After ingestion, EGCG is found primarily in conjugated forms (sulfates and glucuronides) in the plasma, undergoing methylation, glucuronidation, and sulfation, mediated by enzymes such as catechol‐O‐methyltransferase (COMT). Metabolites include 4′‐O‐methyl‐EGCG and dimethyl‐EGCG (Dai et al. 2020; Yang and Wang 2016).

The bioavailability of tea catechins is intrinsically low due to extensive first‐pass metabolism in the small intestine and liver. The limited bioavailability of catechins and their extensive metabolism, particularly in the gastrointestinal tract, requires a more detailed investigation into the role of gut microbiota in facilitating their conversion into bioactive metabolites such as 3,4‐dihydroxyphenylacetic acid (DHPA) and 3‐(3′‐hydroxyphenyl) propionic acid (HPPA), as well as hydroxyphenylvaleric acids and valerolactones (Huang et al. 2013). These metabolites, in turn, influence various physiological pathways, including immune modulation, anti‐inflammatory activity, and the maintenance of gut homeostasis.

Catechin bioavailability is influenced by individual gut microbiota composition. For instance, *Eggerthella lenta* and *Flavonifractor plautii* convert catechins into simpler metabolites, enhancing their bioavailability, though this varies among individuals (Li et al. 2021; Santangelo et al. 2019). Like other catechins, EGCG's low bioavailability is a challenge, with most of it metabolized in the large intestine or excreted. To overcome low bioavailability, nanostructure‐based drug delivery systems and molecular modifications have been proposed. For instance, co‐administering catechins with quercetin or resveratrol has been shown to enhance absorption and prolong their half‐life (Cai et al. 2018; Ottaviani et al. 2016).

### 6.2 Immunomodulatory and Anti‐Inflammatory Activity

Catechin metabolites, particularly short‐chain fatty acids (SCFAs) like acetate, propionate, and butyrate, play a crucial role in regulating immune responses and reducing inflammation. These SCFAs, produced through microbial fermentation of catechins in the colon, promote the growth of beneficial microbes, such as *Faecalibacterium prausnitzii* , and regulate the production of pro‐inflammatory cytokines such as IL‐6, IL‐1β, and TNF‐α (Sun et al. 2021). Moreover, catechin metabolites support the production of regulatory T cells (Tregs), further contributing to immune regulation and the prevention of excessive inflammation (Vernocchi et al. 2020).

Catechins also inhibit key inflammatory pathways, such as MAPK and NF‐κB, reducing immune cell recruitment to inflamed tissues. This helps alleviate chronic inflammation and promote immune resolution (Guo et al. 2019). Catechins' anti‐inflammatory effects have been demonstrated in gastrointestinal diseases like ulcerative colitis and inflammatory bowel disorder. By modulating gut microbiota composition, they promote beneficial bacteria and suppress pathogenic strains, contributing to gut health and immune balance (Sun et al. 2021).

Despite their limited bioavailability and extensive metabolism, catechins demonstrate significant therapeutic potential through their conversion into bioactive metabolites by gut microbiota. These metabolites contribute to immune modulation, inflammation reduction, and gut homeostasis. While bioavailability challenges persist, advances in drug delivery systems offer promising strategies to enhance catechin absorption and efficacy. Continued research into their metabolic pathways and interactions with gut microbiota is crucial to fully harness their therapeutic benefits, particularly in managing chronic diseases. Existing evidence, however, supports a modulatory rather than deterministic role of catechins in maintaining immune balance. The inconsistency between studies underscores limitations such as small sample sizes, limited dietary control, and heterogeneous analytical endpoints. Collectively, these findings illustrate that catechins support immune equilibrium and intestinal health primarily through microbiota‐mediated metabolic conversion and anti‐inflammatory signaling. The mechanisms described here represent preventive or homeostatic processes in healthy systems. How these same pathways become dysregulated or therapeutically targeted during cancer progression is discussed in Section 9.

## 7 Gut Microbiome in Cancer

Cancer is the second leading cause of death globally and is recognized as a multifactorial disease influenced by several interacting factors (Bray et al. 2018; Vivarelli et al. 2019). Among these, the gut microbiota has gained considerable attention because disturbances in its composition are increasingly linked to cancer development (Sadrekarimi et al. 2022; Tao et al. 2020). Gut dysbiosis refers to a disruption of the normal microbial balance in which beneficial microbes are reduced and harmful populations become more prominent, leading to the generation of damaging microbial antigens and metabolites (Asseri et al. 2023; Ge et al. 2021). A key feature of dysbiosis is a reduced *Firmicutes*/*Bacteroidetes* ratio, which is often accompanied by lower SCFAs production and loss of intestinal homeostasis (Pérez‐Burillo et al. 2021). This imbalance may arise due to antibiotic overuse, dietary alterations, infections, inflammatory and chronic diseases, smoking, unhealthy lifestyle habits, and impaired nucleotide‐binding oligomerization domain‐associated responses (Drago et al. 2019; Ge et al. 2021; Sadrekarimi et al. 2022; von Frieling et al. 2018).

Gut microbiota imbalance can influence tumor initiation and progression through multiple interconnected mechanisms. Dysbiosis compromises mucosal barrier integrity, enabling microbial translocation and promoting carcinogenesis. It induces chronic inflammation, causes DNA damage, and leads to the production of carcinogenic metabolites that disrupt cellular homeostasis. Additionally, dysbiotic microbiota release genotoxins that impair genome stability and enhance uncontrolled cellular proliferation while conferring resistance to apoptosis. Tumor development is further facilitated by alterations in key signaling pathways, including β‐catenin/Wnt and other pro‐inflammatory cascades. Moreover, dysbiosis disrupts host immune responses, resulting in immune dysregulation that fosters a tumor‐supportive microenvironment (Ge et al. 2021; Sadrekarimi et al. 2022; Tao et al. 2020; Vivarelli et al. 2019; von Frieling et al. 2018). This interrelationship between the gut microbiota imbalance and cancer risk has been depicted in Figure 4.

**FIGURE 4:**
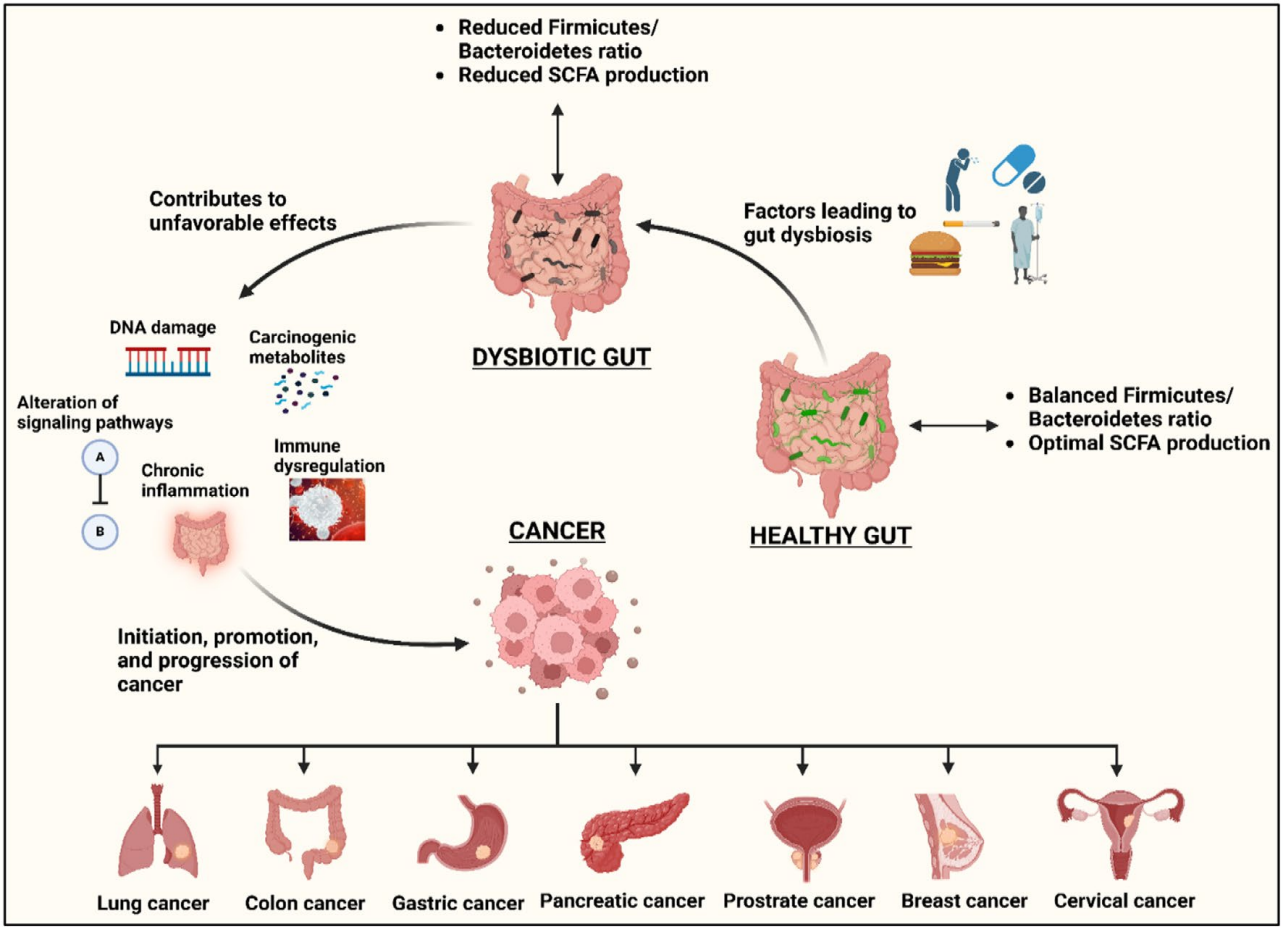
The interrelationship between the gut microbiota imbalance and cancer risk. The shift from a healthy gut to a dysbiotic state driven by factors such as indiscriminate antibiotic use, poor dietary habits, infections, unhealthy lifestyle choices, and autoimmune, chronic, and inflammatory diseases leads to unfavorable effects, in turn leading to potential initiation and promotion of cancer.

The overview of gut microbial shift patterns and associated mechanisms across major cancer types has been represented in Table 3. These dysbiotic alterations represent potential targets for catechins, which have been shown to restore microbial equilibrium, suppress pro‐inflammatory signaling pathways, and enhance the growth of beneficial commensal bacteria. Through their dual antioxidant and microbiota‐modulating activities, catechins may help mitigate the tumor‐promoting effects of dysbiosis while supporting intestinal and systemic homeostasis. The potential implications of catechin‐mediated microbial regulation are elaborated in the following section.

**TABLE 3:** Overview of gut microbial shift patterns and associated mechanisms across major cancer types (↓ denotes decreased abundance; ↑ denotes increased abundance of taxa).

Sl. no.	Cancer type	Altered taxa	Associated mechanisms	References
1	Colorectal cancer	↓ *Faecalibacterium*, *Blautia, Clostridium*, *Bifidobacterium*, *Roseburia*, *Eubacterium* spp. ↑ *Fusobacterium nucleatum* , *E. coli* , *B. fragilis* , *Clostridium symbiosum* , *Enterococcus faecalis* , *Gemella morbillorum* , *Parvimonas micra* , *Shigella*, *Solobacterium moorei* , *Peptostreptococcus stomatis* , *Streptococcus gallolyticus* , *Campylobacter*, *Dialister*, *Malassezia*, *Trichosporon*, *Tunalikevirus*, *Orthobunyavirus*, *Inovirus*	Chronic inflammation; DNA damage; impaired epithelial barrier; genotoxin production; β‐catenin/Wnt activation; SCFA depletion; immune dysregulation	(Chen et al. 2013; Mármol et al. 2017; Nakatsu et al. 2018; Pérez‐Burillo et al. 2021; Sadrekarimi et al. 2022; Sánchez‐Alcoholado et al. 2020; Seesaha et al. 2020)
2	Gastric cancer	↓ *Rhizopus*, *Lecanactis*, *Coprinopsis*, *Exophiala*, *Russula*, *Rhodotorula* ↑ *Lactobacillus*, *Veillonella*, *Prevotella*, *Streptococcus mitis* , *S. parasanguinis* , *Porphyromonas stomatitis*, *Dialister pneumosintes* , *S. exigua* , *S. anginosus* , *Citrobacter*, *Rhodococcus*, *Clostridium*, *Achromobacter*, *Phyllobacterium*, *Prevotella copri* , *Propionibacterium acnes* , *Enterobacteriaceae*, *Malassezia*, *Apiotrichum*, *Pichia*, *Sarocladium*, *Cutaneotrichosporon*, *Chaetomium*	Chronic gastric inflammation; mutagenic metabolite formation; altered stem‐cell dynamics; proliferative signaling	(Abreu and Peek 2014; Asseri et al. 2023; Sarhadi et al. 2021; Wang et al. 2022; Yang et al. 2022)
3	Brain cancer	↓ *Lachnospira*, *Bifidobacterium*, *Firmicutes*, *Actinobacteria*, *Verrucomicrobia* ↑ *Enterobacteriaceae*, *Akkermansia*, *Fusobacterium*, *Proteobacteria*, *Fusobacteriota*, *Escherichia/Shigella*, *Bacteroides*, *Parasutterella*, *Ruminococcus gnavus* group	Microglial dysfunction (M2c activation via IL‐10, TGF‐β); immunosuppression; chronic inflammation; disrupted gut‐brain axis	(Jiang et al. 2022, 2024; Li, Jiang, et al. 2022)
4	Head and Neck cancer	↑ *Selenomonadales*, *Ruminococcus1*, *Dorea*, *Odoribacter*, *Turicibacter*, *Ruminococcaceae* UCG014, *Negativicutes* (class)	Microbial dysbiosis‐driven inflammation; altered mucosal immune response	(Long et al. 2023)
5	Breast cancer	↑ *Clostridium coccoides* cluster, *Bacteroidetes*, *C. leptum* cluster, *Blautia*, *Faecalibacterium prausnitzii* , *Ruminococcaceae*, *Clostridiaceae*	Chronic inflammation; microbial metabolite imbalance	(Luu et al. 2017; Wang et al. 2022)
6	Lung cancer	↑ *Ruminococcaceae* UCG003, *Eubacterium coprostanoligenes* group, *Parasutterella*, *Lactobacillales*, *Clostridium_*XIVa, *Butyricicoccus*	Gut‐lung axis dysregulation; altered immune homeostasis; toxin and virulence‐factor production; genotoxic stress	(Long et al. 2023; Wang et al. 2024)
7	Prostate cancer	↓ *Faecalibacterium prausnitzii* , *Eubacterium rectale* ↑ *Bacteroides massiliensis* , *Lachnospira*, *Rikenellaceae*, *Alistipes*, *Streptococcus*, *Bacteroides*, *Akkermansia*, *Terrisporobacter*, *Roseburia*	Inflammation; immune dysregulation; microbial metabolite‐mediated proliferation	(Golombos et al. 2018; Liss et al. 2018; Long et al. 2023; Matsushita et al. 2021)
8	Cervical cancer	↓ *Bifidobacterium*, *Clostridium*, *Faecalibacterium*, *Ruminococcus*, *Gemmiger*, *Lachnospira*, *Roseburia*, *Bacteroides*, *Blautia*, *Lachnospiraceae* (uncl.), *Clostridiales* (uncl.), *Haemophilus* ↑ *Dialister*, *Porphyromonas*, *Prevotella*, *Actinobaculum*, *Finegoldia*, *Peptostreptococcus*, *Corynebacterium*, *Varibaculum*, *Anaerococcus*, *Streptococcus*, WAL_1855D, *Peptoniphilus*	Chronic inflammation; dysregulated immune response; oncogenic metabolite production	(Kang et al. 2020; Sims et al. 2019)
9	Bladder cancer	↓ *Prevotella*, *Clostridium* cluster XI	Dysbiosis‐driven inflammation and urogenital barrier dysfunction	(Wang et al. 2022)
10	Pancreatic cancer	↑ *Firmicutes*, *Bacteroidetes*, *Actinobacteria*, *Proteobacteria*, *Fusobacteria*, *Verrucomicrobia*, *Prevotella*, *Bifidobacterium*, *Porphyromonas*	Chronic inflammation; microbial metabolite imbalance; altered immune signaling	(Wang et al. 2022)

## 8 Effect of Catechins on Gut Microbiota in Cancer

Catechins have been shown to effectively reverse dysbiosis by promoting the proliferation of beneficial bacteria and simultaneously inhibiting the growth of harmful strains, thereby restoring a healthier balance within the gut microbiota and enhancing overall gut health (Pérez‐Burillo et al. 2021). EGCG, the most prevalent catechin in green tea, making up about 60% of its total catechin content, is recognized as a primary active ingredient in green tea with anti‐cancer properties (Leung et al. 2024; Xu et al. 2020). Animal and cell studies have demonstrated that EGCG exhibits inhibitory effects on various types of cancers (Xu et al. 2020). It has been observed that EGCG increases the abundance of *Lactobacillus* and *Bifidobacterium*, which exhibit counteractive effects on cancer by reducing intestinal inflammation (Torres‐Maravilla et al. 2021; Wang et al. 2022). EGCG promotes the growth of bacteria that support the synthesis and metabolism of SCFAs and phenolic acids. Alternatively, it reduces the population of bacteria that produce harmful metabolites like bacterial toxins, pro‐inflammatory cytokines, polyamines, hydrogen sulfide, and secondary bile acids. This positively influences the gut microbiota, reducing the risks associated with colorectal cancer, hepatocellular carcinoma, and lung cancer (Leung et al. 2024). EGCG also boosts the *Bifidobacterium* level, which subsequently enhances the production of SCFAs; this further inhibits inflammatory pathways activated by NF‐κB that would otherwise facilitate prostate cancer carcinogenesis (Kumar et al. 2022). The impact of EGCG on gut microbiota balance and cancer prevention has been depicted in Figure 5.

**FIGURE 5:**
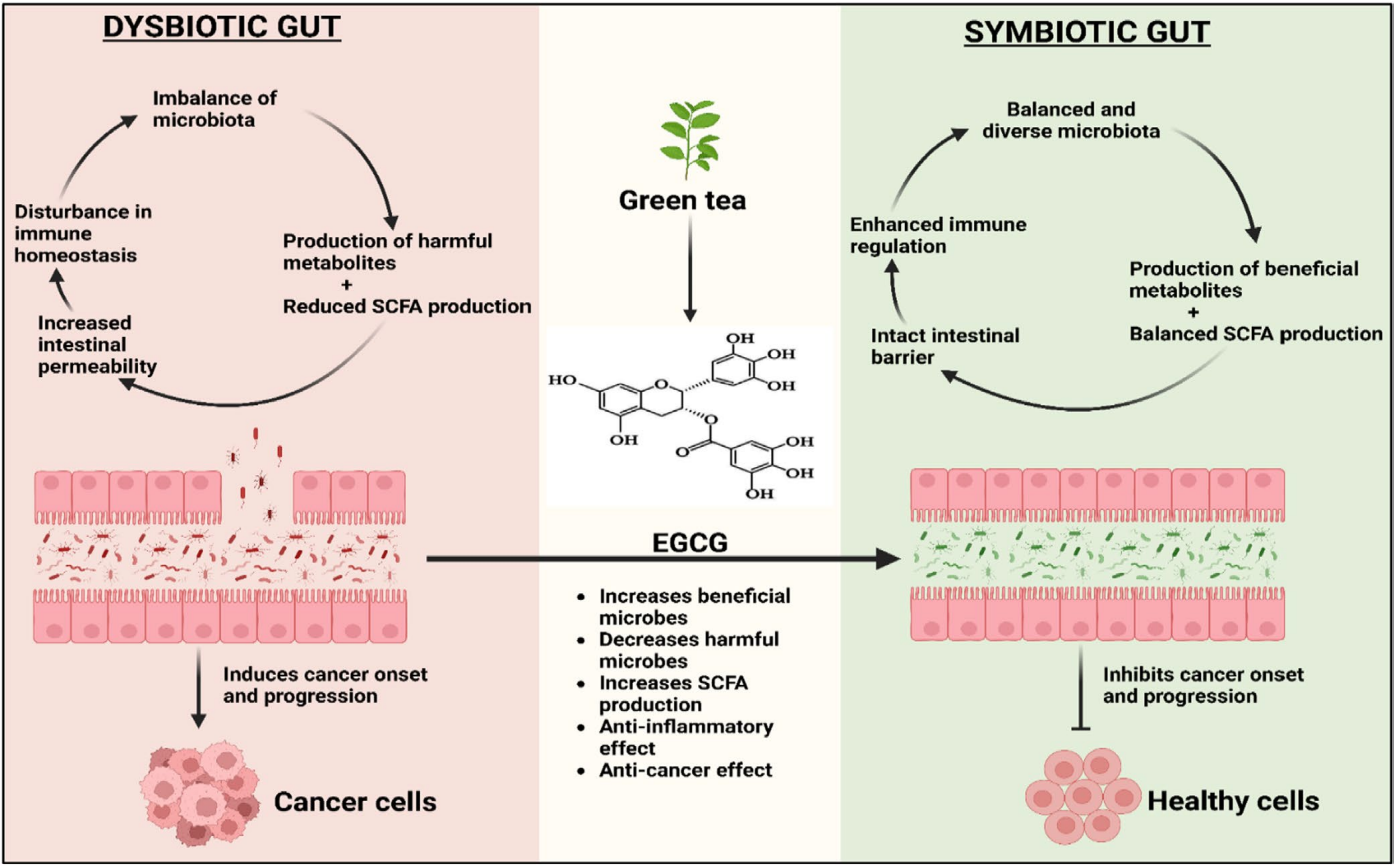
Impact of EGCG on gut microbiota balance and cancer prevention. EGCG, a key catechin present in green tea, has the potential to shift the gut microbiome from dysbiosis to symbiosis, thereby enhancing the gut health and inhibiting cancer progression.

A study was performed by Li et al. on C57BL/6J mice subjected to a high‐fat diet alongside a multi‐dose urethane treatment to induce lung tumorigenesis. It was observed that these induced conditions altered the gut microbiota. However, treatment with EGCG reversed the effect by reducing the prevalence of *Clostridia* and raising the prevalence of *Deltaproteobacteria* and *Epsilonproteobacteria* (Li, Hao, et al. 2023). In female FVB/N mice with colon cancer induced by azoxymethane‐dextran sodium sulfate, EGCG treatment rebalanced the gut microbiota disrupted by the disease. The abundance of beneficial bacteria such as *Fusobacterium*, *Prevotella*, *Desulfococcus*, *Veillonella*, *Ruminococcus*, *Lactobacillus*, *Clostridiaceae*, *Enterobacteriaceae*, *Sulfurimonas*, and *Ochrobactrum* was increased. Meanwhile, there was a reduction in harmful bacteria like *Bacteroides*, *Anaerotruncus*, and *Faecalibaterium* (Zhao and Jiang 2021). EGCG, the predominant catechin in green tea, has demonstrated significant anticancer properties. Collectively, EGCG inhibits carcinogenesis by stabilizing the gut microbiota, hindering pathogenic bacterial growth, and by fostering the expansion of beneficial probiotics. Thus, EGCG may serve as a valuable dietary component in cancer management strategies. However, further research is required to thoroughly map these changes and develop optimal dietary strategies employing other catechins alongside EGCG for effective cancer management.

## 9 Mechanism of Action of Catechins on Gut Microbiota in Cancer

Building upon the mechanisms described in Section 6, which outlines the homeostatic roles of catechins in healthy individuals, this section focuses on their functions in pathological conditions, specifically cancer. Under tumor‐associated dysbiosis and chronic inflammation, catechins influence key signaling pathways that govern cell proliferation, apoptosis, and immune modulation. Understanding these altered mechanisms clarifies how catechins contribute to cancer prevention and therapy. Following the in vivo investigations of catechins' effects on gut microbiome composition, physiology, metabolism, and biotransformation in cancer conditions, a closer examination of the molecular mechanisms underlying these interactions is warranted. The therapeutic potential of catechins is rooted in their ability to modulate critical signaling pathways involved in cancer progression, immune responses, and cellular proliferation. By elucidating the specific mechanisms through which catechins exert their anticancer effects, a more comprehensive understanding of their contribution to cancer prevention and treatment can be established. Despite the development of numerous therapeutic approaches, the adverse effects of standard treatments have driven the search for natural alternatives. Among these, green tea catechins have garnered significant attention due to their favorable physiological effects (Cheng et al. 2020).

Both in vitro and in vivo studies have consistently demonstrated the antiproliferative properties of green tea extracts. Polyphenols found in green tea have demonstrated the ability to suppress the growth of cancer cells in several types, including breast, lung, colorectal, prostate, lymphoma, and leukemia. Furthermore, prolonged consumption of green tea extracts has been associated with enhanced activity of glutathione S‐transferase (GST), an essential enzyme for detoxification, as well as inhibition of angiogenesis, thereby reducing tumor metastasis by inhibiting the formation of new blood vessels (Przystupski et al. 2019).

Cancer types exhibit considerable heterogeneity in terms of genetic profiles, treatment approaches, and prognosis. These variations are linked to specific hallmarks of cancer, which can be targeted by catechins through diverse mechanisms. Catechins modulate cancer cell proliferation by regulating pathways such as the MAPK/ERK involved in sustaining proliferative signaling, as well as disrupting p53‐dependent growth suppression. Additionally, catechins inhibit immune evasion by modulating cytokine secretion (Cadoná et al. 2022). Tumor necrosis factor‐alpha (TNF‐α), a critical pro‐inflammatory cytokine associated with multiple cancers, is crucial for cancer progression. The downregulation of TNF‐α through the inhibition of NF‐κB is essential in mitigating cancer development, as depicted in Figure 6. Yet, some investigations have observed minimal or even opposing responses, reflecting the dual antioxidant–pro‐oxidant nature of catechins and the differing redox environments of specific tumor models (Khan and Mukhtar 2019).

**FIGURE 6:**
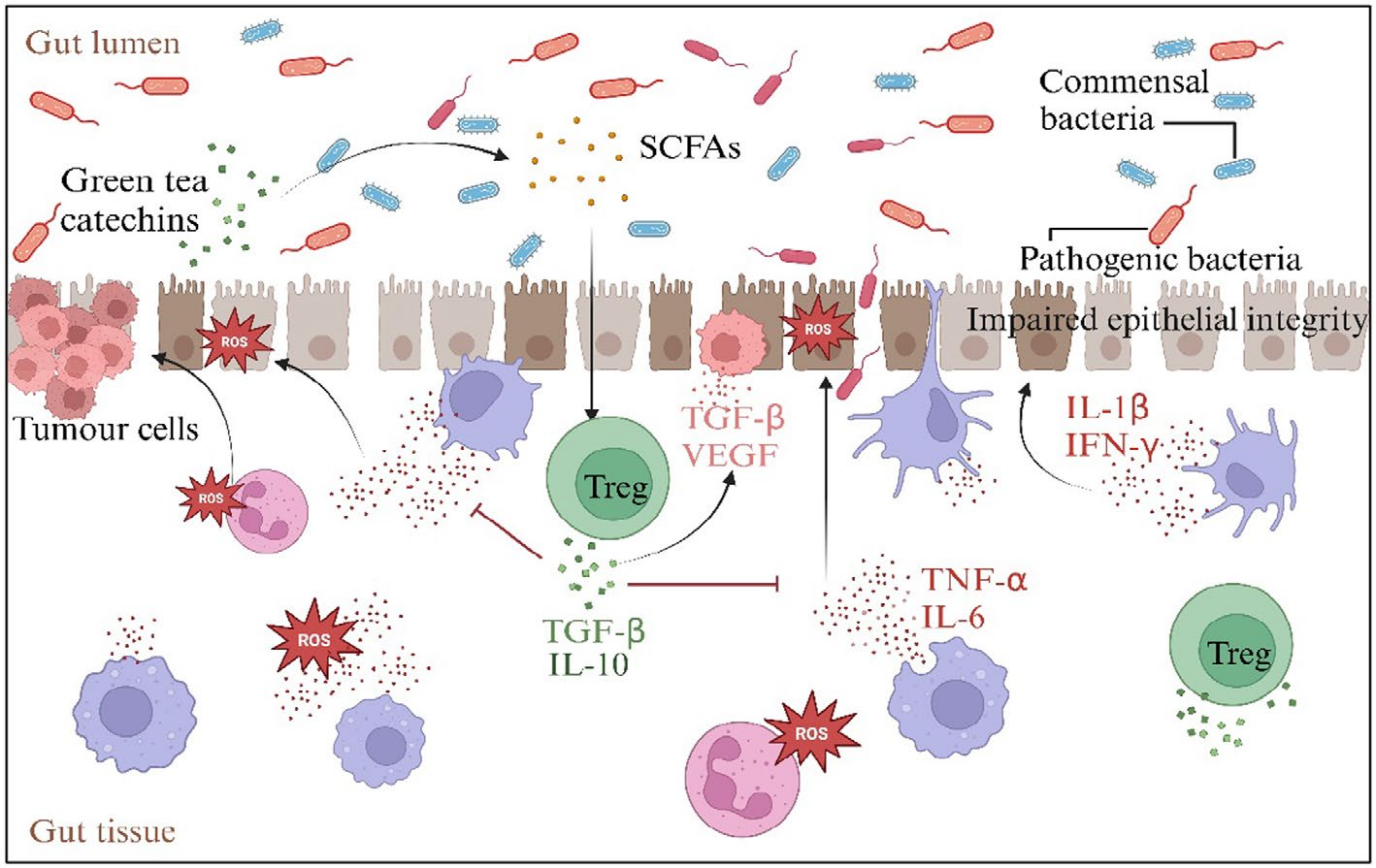
Mechanism of action of catechins on the gut microbiota in cancer. This figure illustrates the altered interaction between green tea catechins and the gut microbiota in a tumor‐associated environment. Despite the presence of green tea catechins, tumor cells and reactive oxygen species (ROS) contribute to impaired epithelial integrity and increased oxidative stress. Commensal bacteria continue to produce SCFAs; however, pathogenic bacteria proliferate, exacerbating epithelial damage and inflammation. The disrupted barrier allows for an enhanced inflammatory response, driven by cytokines such as IL‐1β and IFN‐γ. Regulatory T cells (Tregs) still attempt to exert an anti‐inflammatory effect through the secretion of TGF‐β and IL‐10, but tumor cells simultaneously release VEGF, fostering a pro‐tumor microenvironment. The figure highlights the dynamic interplay between the gut microbiota, immune cells, and tumor cells, which collectively influence the inflammatory state during tumor progression.

EGCG, a predominant green tea catechin, has been shown to exert potent anti‐inflammatory effects, including TNF‐α inhibition and protection against TNF‐α‐induced lung inflammation by reducing oxidative stress and ICAM‐1 expression. Additionally, EGCG suppresses TNF‐α‐induced MCP‐1 production in endothelial cells and selectively blocks COX‐2 without impacting COX‐1 in prostate cancer cells, highlighting its potential use in combination with chemotherapeutic agents (Almatroodi et al. 2020). The ability of catechins to target various hallmarks of cancer, including resisting cell death, sustaining proliferative signaling, evading growth suppressors, and tumor‐promoting inflammation, demonstrates their potential as promising anti‐cancer agents as shown in Figure 7.

**FIGURE 7:**
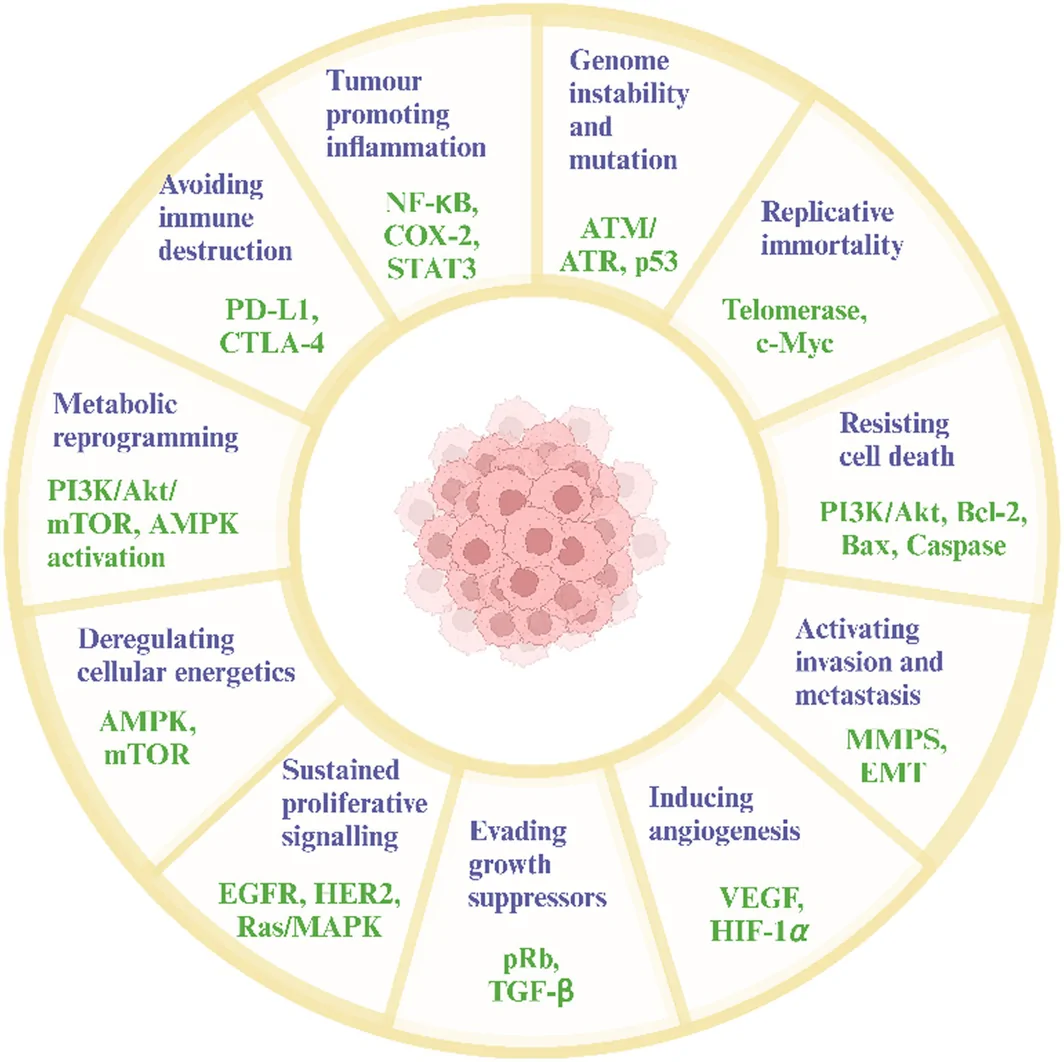
Therapeutic targets of catechins in cancer hallmarks. This figure depicts the hallmark characteristics of cancer cells, each supported by key molecular pathways. Genome instability and mutation are driven by alterations in ATM, ATR, and p53, while replicative immortality is maintained via telomerase and c‐Myc. Cancer cells evade cell death through the PI3K/Akt pathway, Bcl‐2, and caspase inhibition, and invade/metastasize through MMPs and EMT. Angiogenesis is promoted by VEGF and HIF‐1α, while immune evasion occurs through PD‐L1 and CTLA‐4. Cancer progression is supported by inflammation via NF‐κB and COX‐2, metabolic reprogramming by PI3K/Akt and AMPK, and proliferation through EGFR and HER2 signaling, with TGF‐β facilitating the evasion of growth suppressors.

The selective anticancer effects of catechins and other plant‐based polyphenols are largely attributed to their prooxidant activities, which are heightened in malignant cells due to elevated copper levels compared to non‐malignant cells (Farhan et al. 2022). Moreover, histones and histone deacetylases play critical roles in cancer cell proliferation. SIRT1, a member of the Class III histone deacetylases, is particularly significant, and galloylated catechins like EGCG inhibit SIRT1 protein expression, thereby promoting apoptosis and halting cancer cell proliferation (Jiang et al. 2022).

Regulation of matrix metalloproteinases (MMPs), enzymes involved in metastasis, is another crucial factor in cancer management. Catechins, particularly EGCG, have been shown to suppress β‐catenin oncogenic signaling, which plays a key role in cancer progression. Additionally, catechins influence chemopreventive effects through the ERK and p38MAPK signaling pathways (Zhou et al. 2016). Beyond gene expression modulation, catechins also impact cell cycle regulation. They induce cell cycle arrest at the G1 phase by modulating key regulators such as p21/WAF1/CIP1, p27/KIP1, cyclin D1, cdk4, and cdk6. Notably, catechin pyrogallol has been reported to inhibit the G2‐M phase transition, contributing to tumor growth suppression (Li, Feng, et al. 2022).

The multifaceted effects of catechins on cancer biology underscore their therapeutic promise as natural alternatives to conventional treatments. By targeting various signaling pathways, modulating immune responses, and regulating cell cycle dynamics, catechins not only demonstrate significant antiproliferative properties but also highlight their potential in enhancing existing therapeutic strategies. Overall, while mechanistic findings are compelling, they remain constrained by variations in model design, catechin formulation, and absence of standardized exposure metrics. Integrating microbiome‐humanized animal models and quantifying actual tissue concentrations could reconcile these discrepancies.

## 10 Conclusions

Catechins maintain health by exerting preventive and restorative effects, such as enhancing gut microbiota diversity and supporting anti‐inflammatory pathways. In cancer, however, these same mechanisms are redirected toward therapeutic outcomes suppressing tumor‐promoting inflammation, disrupting proliferative signaling pathways like NF‐κB and MAPK, and inducing apoptosis. Collectively, evidence highlights that the biological activity of catechins is context‐dependent, shaped by dosage, formulation, individual microbiota composition, and host redox state. This explains the variability and inconsistent outcomes across studies and reinforces that catechins act as modulators rather than universally beneficial compounds. Ultimately, catechins, particularly EGCG, hold considerable promise as modulators of gut–host homeostasis and as complementary therapeutic agents in cancer prevention and treatment.

## 11 Research Gaps

Despite promising mechanistic data, translation into clinical applications remains challenging. Catechins have poor stability and low bioavailability, and their effects vary significantly due to inter‐individual differences in microbial biotransformation and metabolism. Additionally, most current insights are derived from in vitro or animal studies that do not fully replicate the complexity of the human microbiome–immune interface. This methodological gap limits predictive accuracy and restricts clinical relevance.

## 12 Future Developments

To overcome these challenges, future research should focus on systematic dose–response investigations using physiologically achievable concentrations. Integrating metabolomics and microbiome analyses is essential to map host–microbe co‐metabolism and identify predictive biomarkers of responsiveness. Advanced delivery platforms such as nanoencapsulation or molecular conjugates should be developed to improve catechin stability, bioavailability, and tissue targeting. Equally important are well‐controlled human clinical trials that stratify participants based on baseline microbiota composition to enhance reproducibility and individualized therapeutic predictions. Addressing these priorities will enable mechanistically grounded and clinically translatable strategies for catechin‐based interventions.

## Author Contributions

**Prakash Monika:** validation, supervision, writing – review and editing. **Maitreyi Darbha:** writing – original draft. **Bindu Sadanandan:** conceptualization, validation, supervision, writing – review and editing. **Srinivas Reneeka:** writing – original draft. **Kirti Gupta:** writing – original draft. **Honnamachanahalli Ramachandraiah Tejashree:** writing – original draft.

## Conflicts of Interest

The authors declare no conflicts of interest.

## Data Availability

The data that support the findings of this study are available from the corresponding author upon reasonable request.
